# Metabolomics- and systems toxicology-based hepatotoxicity mechanism of *Sophorae Tonkinensis Radix et Rhizoma* in rats

**DOI:** 10.3389/fphar.2022.1015008

**Published:** 2022-11-18

**Authors:** Dengxiang Yu, Zhen Shao, Yuemeng Fu, Xiaohang Tang, Qilong Chen, Zhongping Deng

**Affiliations:** ^1^ Innovation Research Institute of Traditional Chinese Medicine, Shanghai University of Traditional Chinese Medicine, Shanghai, China; ^2^ Shanghai Skin Disease Hospital, School of Medicine, Tongji University, Shanghai, China

**Keywords:** sophorae tonkinensis radix et rhizoma, alkaloids, hepatotoxicity, metabolomics, systems toxicology, bile acid metabolism

## Abstract

Drug-induced liver injury (DILI) is a major challenge to the development and clinical application of drugs, especially limits the global application of Chinese herbal medicines, because the material basis and mechanisms of some Chinese herbal medicines are not well clear. In this study, a comprehensive method integrating metabolomics and systems toxicology (SysT) was used to investigate how the main substances in *Sophorae Tonkinensis* *Radix et Rhizoma* (STRER) influence the metabolic pathways and molecular mechanisms of hepatotoxicity. Through a 28-day continuous oral administration toxicity study combined with serum metabolomics analyses, the aqueous, ethanol-precipitation and dichloromethane extracts of STRER exhibited significant hepatotoxic effects. In addition, 19 differential metabolites with a time-dose-effect relationship were identified in rats. The primary bile acid biosynthesis pathway was significantly altered, which was consistent with the findings of the SysT analysis. Furthermore, through the quantification of bile acids in serum, 16 differential bile acids were identified as being significantly changed; moreover, 21 relevant targets which intersected with the hepatotoxic targets of STRER were identified. Molecular docking was used to confirm the validation of bindings between targets and corresponding compounds, and finally, six important compounds and 14 potential targets were identified to be involved in STRER-induced liver injury in relation to bile acid metabolism.

## 1 Introduction

*Sophorae Tonkinensis Radix et Rhizoma* (STRER), commonly known as “Shan-Dou-Gen,” is a traditional Chinese medicine (TCM) which has heat-clearing, detoxifying, edema-alleviating, and sore throat-relieving effects (Chinese Pharmacopoeia Commission, 2020). Alkaloids are the main active ingredients in STRER, among which matrine (MT) and oxymatrine (OMT) are considered its quality index components. STRER alkaloids have been shown to exhibit diverse pharmacological effects, including anti-tumor, anti-inflammatory and antibacterial effects (Gotti and Clementi, 2021; Sun et al., 2022; Tang et al., 2022). However, the toxic effects of STRER alkaloids, which include liver injury, have significantly restricted its clinical use and development (Lu et al., 2014; Liu et al., 2017; Gu et al., 2019).

Drug-induced liver injury (DILI) is caused by drugs or other xenobiotics following toxic-dose exposure; it is classified into three types, intrinsic, idiosyncratic, and indirect injury (Andrade et al., 2019; Hoofnagle and Björnsson, 2019). Chinese herbal medicines have been reported to be partly responsible for the increasing DILI incidence in China, owing to the large population of the country and their easy access to a wide range of drugs (European Association for the Study of the Liver, 2019). A high oral dose of STRER water extract was found to induce acute liver injury, and long-term administration cumulative poisoning with this extract induced liver toxicity in a dose-dependent manner (Wang et al., 2017). The alkaloid-rich STRER extract was also found to exhibit a dose-response hepatoxicity relationship (Liu et al., 2017). Omics approaches, including genomics, transcriptomics, proteomics, and metabolomics, are effective research tools for investigating the mechanisms underlying DILI (Kralj et al., 2021). Metabolomics is a systematic qualitative and quantitative analysis tool that can be used to investigate endogenous metabolites, determine DILI phenotypes, understand the dynamic development and recovery process of DILI, and provide standard biomarkers for clinical use before physiological or pathological damages appear (Holmes et al., 2008; Liu et al., 2018; Xie et al., 2019). Recently, changes in the levels of free bile acids, conjugated bile acids, and glycerol phospholipids were shown to be the most relevant metabolic features of DILI phenotypes (Ma et al., 2019, Quintás et al., 2021). The complex composition, multi-target and multi-pathway mechanisms of herbal medicines are the main difficulties in toxicity studies on TCMs. The mechanism of herbal medicine-induced DILI cannot be completely determined using a single research method (Pelkonen et al., 2014). In recent years, computational toxicology, as well as bioinformatics methods and molecular toxicology, have emerged as novel strategies for research on the toxicity mechanisms of TCMs (Gao et al., 2019). SysT was applied to investigate the toxicity of compounds and describe their toxic effects, based on the “toxic compounds-targets-pathways” network, by integrating systems biology and network analysis (Kiani et al., 2016; Lee et al., 2019). SysT in combination with multi-omics analyses has broadened research avenues in drug toxicity research; and metabolomics was combined with SysT to screen for differential metabolites to systematically and detailly describe the substance basis and intrinsic toxicity mechanism of TCMs (Li et al., 2019; Liu et al., 2019; He et al., 2021).

To reveal the hepatotoxic effect and molecular mechanism of STRER, metabolomics was used to discover the metabolites with significant changes from an *in vivo* metabolic environment of STRER. Systems toxicology was integrated to reveal the key targets of STRER in bile acid metabolism using the network mode of “compounds-hepatotoxic targets-bile acids”, and the hepatotoxic targets inducing aberrant bile acid metabolism were verified by molecular docking methods. We try to elucidated the differential serum bile acids and possible mechanisms of STRER in liver injury, which might offer some important insights into scientific research and clinical applications.

## 2 Materials and methods

### 2.1 Chemicals and reagents

Sulfuric acid (Lot: 20091117), 95% ethanol (Lot: 20160929), dichloromethane (Lot: 20160506), and sodium hydroxide (Lot: 20141017) were purchased from Sinopharm Chemical Reagent Co., Ltd. (Shanghai, China). MT (Lot: 110805–200508) and OMT (Lot: 110780–201508) were purchased from the Chinese National Institute for the Control of Pharmaceutical and Biological Products (Beijing, China). HPLC grade acetonitrile and methyl alcohol were purchased from Merck (Darmstadt, Germany) and formic acid was purchased from CNW Technologies (Duesseldorf, Germany). Kits for determining alanine aminotransferase (ALT), aspartate aminotransferase (AST), alkaline phosphatase (ALP), and total bilirubin (TBIL) levels were purchased from Shino-Test Corporation (Tokyo, Japan). Kit for total bile acid (TBA) was purchased from AusBio Laboratories Co., Ltd. (Yantai, China). Tauroursodeoxycholic acid (TUDCA), taurohyodeoxycholic acid (THDCA), taurocholic acid (TCA), glycoursodeoxycholic acid (GUDCA), glycocholic acid (GCA), taurochenodeoxycholic acid (TCDCA), taurodeoxycholic acid (TDCA), ursodeoxycholic acid (UDCA), hyodeoxycholic acid (HDCA), glycochenodeoxycholic acid (GCDCA), glycodeoxycholic acid (GDCA), cholic acid (CA), taurolithocholic acid (TLCA), chenodeoxycholic acid (CDCA), deoxycholic acid (DCA), lithocholic acid (LCA) and mycophenolic acid were purchased from Sigma-Aldrich Trading Co., Ltd. (Shanghai, China).

### 2.2 Preparation of STRER extracts

*Sophorae Tonkinensis Radix et Rhizoma* (Lot: 1610058) was purchased from Sichuan Neautus Traditional Chinese Medicine Co., Ltd. (Chengdu, China). The dried STRER was soaked in water for 30 min and extracted thrice at 100°C for 1 h (1:6, w/v). The integrated filtrate was concentrated under vacuum at 60°C to a relative density of 1.15 g/mL. A quarter of the concentrated solution was dried to obtain the aqueous extract (AE). To the rest of solution, 95% ethanol was added and stirred to obtain an 80% ethanol mixture. Then, the sediment fraction was collected after allowing it to stand overnight at 20°C, which was dried at 60°C, crushed and passed through a 40 mesh sieve to obtain ethanol-precipitation extract (EPE). Next, ethanol of the supernatant was removed and 2% sulfuric acid was added to the solution to regulate the pH (4–5). The pH of the filtrate was adjusted to the 10–11 range using a NaOH solution after leaving it to stand for 3.5 h; extraction was then performed thrice on this filtrate using dichloromethane. After recovering the solvent, the extract was dried under vacuum at 60°C to obtain the dichloromethane extract (DCME).

### 2.3 HPLC detection of MT and OMT in STRER extracts

Chromatographic analysis was performed on the Agilent 8453 Series High-Performance Liquid Chromatography system (Agilent Technologies, California, United States). Chromatographic separation was carried out using a Waters XBridge C18 column (4.6 × 250 mm, 5 μm, Waters, Massachusetts, United States) at 30°C and a mobile phase constituted of acetonitrile (A) and 0.1% formic acid (B). UV absorption was detected at 210 nm with isocratic elution of 5% A. The flow rate was set at 1.0 mL/min. The standard calibration curves for MT and OMT were prepared using concentrations ranging from 37.5 to 600 and 25 to 400 μg/mL, respectively. The linear regression equation and correlation coefficient were derived from the graph between the concentration and peak area of the standard solution. STRER extracts dissolved in the mixed mobile phase solution at a concentration of 1 mg/mL were filtrated through 0.22-μm microporous membranes, and 10 μL of the filtrate was injected into the HPLC system for quantitative analysis.

### 2.4 Systems toxicological analysis

Based on the fact that the oral route of administration is commonly used in TCM, compounds in STRER were screened in the Traditional Chinese Medicine Systems Pharmacology Database and Analysis Platform (TCMSP, https://www.tcmsp-e.com/) using important pharmacokinetic parameters such as drug likeness (DL) > 0.15 and oral bioavailability (OB) > 50%. Hepatotoxic targets were searched in the Genecards database (https://www.genecards.org/) using “liver toxicity” as keyword, with relevance score≥10. The targets of STRER constituents and differential bile acids were obtained from the SwissTargetPrediction platform (http://www.swisstargetprediction.ch/). Gene names and ontology annotations were extracted from the UniProt database (https://www. uniprot. org/). The hepatotoxic targets of STRER compounds were extracted using Cytoscape 3.9.1 (https://cytoscape.org/). Protein-protein interaction (PPI) analysis data obtained from the IntAct (https://www.ebi.ac.uk/intact/), BioGRID (https://thebiogrid.org/), and MINT (https://mint.bio.uniroma2.it/) databases were transported to Cytoscape for the construction of the STRER-induced hepatotoxic targets interaction network. The network was filtered by betweenness centrality (BC)≥avg (BC), closeness centrality (CC)≥avg (CC), and degree (De)≥avg (De) to obtain the potential hub nodes of STRER-induced hepatotoxicity. Subsequently, GO function and KEGG pathway analyses were conducted using Metascape (https://metascape.org/).

### 2.5 Animal experiment

#### 2.5.1 Animals and sample collection

SPF-grade Wistar rats of both sexes (equal numbers) with an average body weight of 110 ± 10 g were purchased from Shanghai SLAC Laboratory Animal Co., LTD. (Shanghai, China) (animal license No. SCXK (Hu) 2012–0002). The rats were housed in Shanghai University of Traditional Chinese Medicine Laboratory Animal Center (animal license No. SYXK (Hu) 2014–0008), under controlled environmental conditions (20–25°C, 50–70% relative humidity, 12-h light-dark cycle). All animal experiments were approved by the Shanghai University of Traditional Chinese Medicine institutional Animal Care and Use Committee. All procedures were conducted in accordance with the Chinese national legislation and local guidelines, following the principles of animal welfare.

Following acclimatization for 1 week, the rats were randomly divided into three time point groups (Day 7, Day 14, and Day 28). Each time point was further divided into eight groups (*n* = 10, five male and five female rats), the control group, in which rats were administered pure water, the DCME-L group (20 mg/kg, equivalent to 3.5 g/kg STRER), the DCME-M group (80 mg/kg, equivalent to 14 g/kg STRER), the DCME-H group (240 mg/kg, equivalent to 42 g/kg STRER), the EPE-L group (0.275 g/kg, equivalent to 3.5 g/kg STRER), the EPE-M group (1.1 g/kg, equivalent to 14 g/kg STRER), the EPE-H group (3.3 g/kg, equivalent to 42 g/kg STRER) and the AE group (2.91 g/kg, equivalent with 14 g/kg STRER). The rats were administered these treatments repetitively by gavage for 7, 14, and 28 days. The body weights and food consumption of the rats were recorded weekly.

On days 8, 15, and 29, serum samples were collected from the supernatants of rat blood samples following centrifugation at 5000 rpm for 15 min and stored at −80°C for biochemical and metabolomics analyses. Rat liver tissues were collected, weighed, and immediately immersed in 10% formalin for pathological examination. The liver coefficient and liver/brain ratio were calculated using the formulae, liver coefficient (%) = liver weight (g)/animal weight (g)×100% and liver/brain ratio = liver weight (g)/brain weight (g).

#### 2.5.2 Biochemical detection and histopathology analysis

Serum ALT, AST, ALP, TBA, and TBIL levels were determined using an 7080 automatic biochemical analyzer (Hitachi, Kyoto, Japan). Rat liver tissues were stained by hematoxylin-eosin (HE) staining and pathological manifestations were observed under a light microscope to evaluate STRER-induced liver damage.

### 2.6 Metabolomics analysis

#### 2.6.1 Sample preparation

For this analysis, 100-μL serum samples were mixed with 400 μL of methanol and 5 μL of the internal standard (0.3 mg/mL 2-chlorophenylalanine). Then, this mixture was vigorously vortexed and the supernatant was collected for analysis following centrifugation at 12,000 rpm for 15 min at 4°C. Quality control (QC) sample containing the biological information of all samples were prepared by mixing 5 μL of all preprocessed serum samples.

#### 2.6.2 UPLC-LTQ-orbitrap-MS analysis

Samples were separated on a Ultimate 3000LC liquid chromatography system (Thermo Fisher Scientific, Massachusetts, United States) and Orbitrap Elite high-resolution mass spectrometer system (Thermo Fisher Scientific, Massachusetts, United States) using a Waters ACQUITY UPLC T3 column (2.1 mm × 100 mm, 1.7 μm, Waters, Massachusetts, United States) at 40°C and a mobile phase constituted of 0.1% formic acid (A) and 0.1% formic acid in acetonitrile (B). This was carried out using a multistep gradient elution program with the following parameters: 5% B (0–2.0 min), 5–95% B (2.0–12.0 min), 95% B (12.0–15.0 min), and 95–5% B (15.0–17.0 min). The injection volume was 5 μL and the flow rate was set at 0.3 ml/min. Mass spectral analysis was carried out using electrospray ionization (ESI) in positive and negative ionization modes. MS parameters were set as, electrospray voltage: 3800 V under the positive mode and 3200 V under the negative mode; aux gas heater temperature: 300°C; capillary temperature: 350°C; S-Lens RF Level: 30% under the positive mode and 60% under the negative mode; flow rate for sheath and auxiliary gas were set at 45 and 15 arb, respectively.

### 2.7 Bile acid analysis

#### 2.7.1 Sample and standard solution preparation

Activated carbon was added to the sera of rats in the control group at a concentration of 100 mg/mL, vortexed for 1 min, and the supernatant was collected following centrifugation at 12,000 rpm for 15 min after leaving the mixture to stand overnight at 4°C; these steps were repeated thrice. CA, CDCA, DCA, GCA, GCDCA, GDCA, GUDCA, HDCA, LCA, TCA, TCDCA, TDCA, and THDCA were accurately weighed, dissolved, and diluted with methanol to prepare series standard solutions. The 5-μL series standard solutions and 300 μL of IS (Mycophenolic acid at a concentration of 160 ng/mL) were mixed with 100 μL of pre-treated control mouse sera to obtain final standard solutions at concentrations ranging from 5 to 5000 ng/mL. Three hundred microliters of the internal standard was added to 100 μL of serum samples obtained from rats in the experimental groups and the supernatant of this mixture was collected and transferred to an autosampler vial for quantitation after vertexing and centrifugation at 12,000 rpm for 10 min.

#### 2.7.2 UPLC/TQ -MS analysis

Chromatographic separation was carried out using a Waters ACQUITY UPLC C18 column (2.1 mm × 100 mm, 1.7 μm, Waters, Massachusetts, United States) at 45°C on a Waters ACQUITY UPLC instrument (Waters, Massachusetts, United States). Linear gradient elution using a 5-mM ammonium acetate solution in 0.1% formic acid (A) and methanol (B) was carried out as follows: 45% A (0–1 min), 45–20% A (1–9 min), 20–10% A (9–14.1 min), 10–45% A (14–14.1 min), and 45% A (14.1–17 min). Five microliters of the sample was injected into the UPLC system at a flow rate of 0.3 mL/min. The API 5500 triple-quadrupole mass spectrometer (Applied Biosystems, California, United States) with ESI in negative ion and multiple reaction monitoring (MRM) mode was used for mass spectral analysis. The MS parameters were set as follows, capillary voltage: 2800 V; cone hole voltage: 55 V; ion source temperature: 120°C; solvent removal temperature: 350°C; the flow rates of the desolvation (N2) and cone (N2) gases were set at 600 and 5 L/h, respectively.

### 2.8 Molecular docking

The intersection between the targets of the components of STRER and differential bile acids was determined using Venn diagrams (https://bioinformatics.psb.ugent.be/webtools/Veen/) for the construction of the “compounds-hepatotoxic targets-bile acids” network. Related compounds and targets were investigated through molecular docking using AutodockTools 1.5.7 (The Scripps Research Institute, California, United States). The 3D structures of the components of STRER and the crystal structures of key targets were derived from the PubChem database (https://www.ncbi.nlm.nih.gov) and the RCSB Protein data bank (https://www.rcsb.org/). Then, these structures were dehydrated, separated from the ligands, and imported into the AutoDockTools for hydrogenation, gasteiger computation, assigning of atom types, and molecular docking assessment. Finally, the docking model with the best-fit binding energy score was visualized using PyMoL 2.2.0 (DeLano Scientific LLC, California, United States) and used to calculate hydrogen bond lengths.

### 2.9 Data analysis

Experimental data were expressed as “mean ± standard deviation.” Data analysis was carried out using SPSS 21.0 software (SPSS Inc. Chicago, IL, United States) and *p*-values<0.05 were considered statistically significant. Compound Discoverer 2.0 (Thermo Fisher Scientific Inc.Waltham, Massachusetts, United States) was used to calibrate and integrate metabolomics data. Multivariate statistical analysis was performed using Simca-P 13.0 (Umetrics, Umea, Sweden). The Metaboanalyst platform (https://www.metaboanalyst.ca/) was used to analyze the pathways of differential metabolites.

## 3 Results

### 3.1 Systems toxicological analysis for hepatotoxic targets and pathways of STRER

**FIGURE 1:**
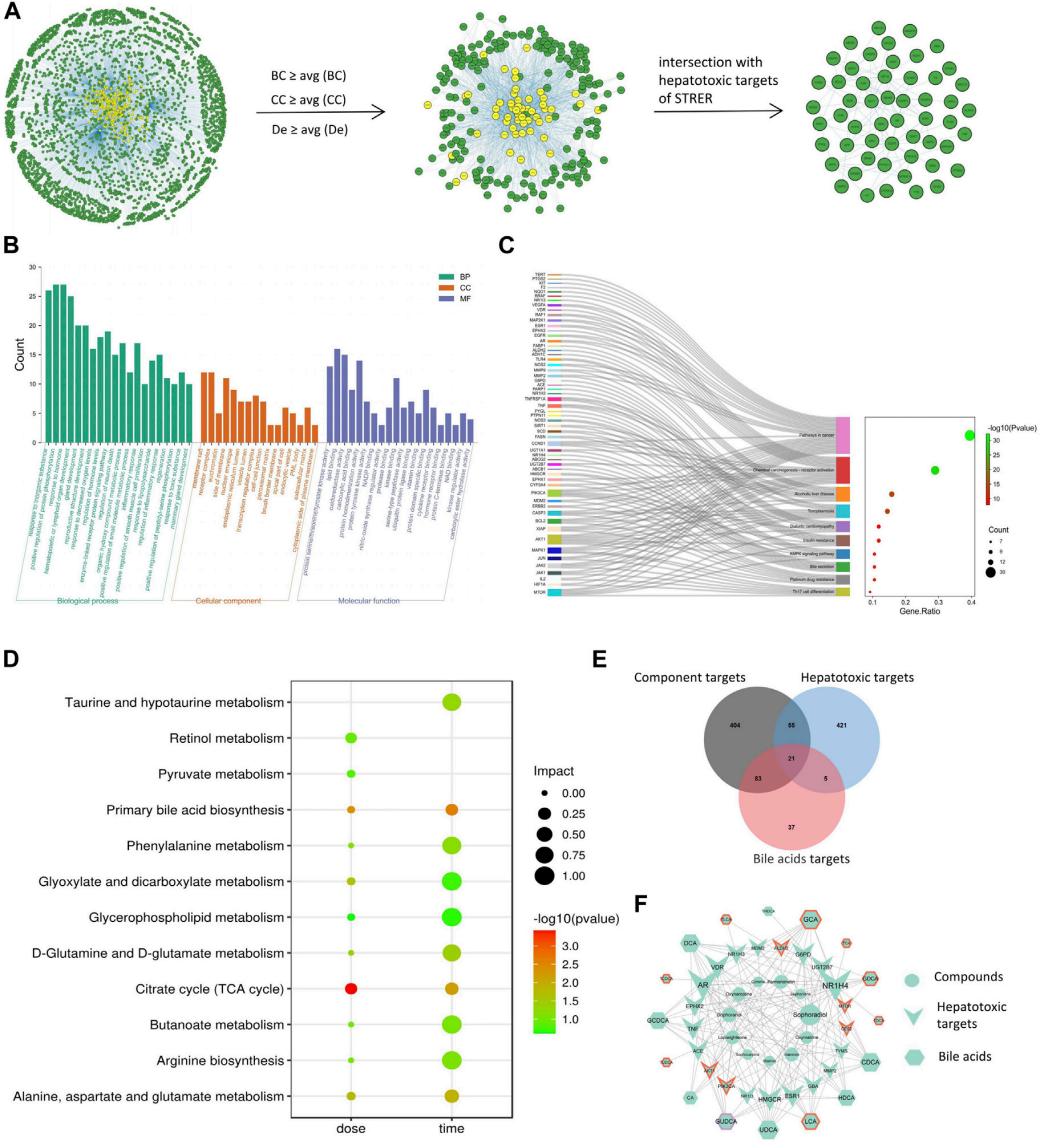
Net analysis of STRER hepatotoxic substance basis, target and metabolites based on SysT. **(A)** PPI network of STRER hepatotoxic targets and hub nodes screened by network parameters. **(B)** GO analysis in three terms including biological process (BP), cellular component (CC), and molecular function (MF). **(C)** KEGG analysis of STRER hepatotoxic targets. **(D)** KEGG pathway enrichment analysis of differential metabolites from non-targeted metabolomics. **(E)** Venn diagram of intersection genes among STRER hepatoxic components and bile acid targets. **(F)** The “compounds-hepatotoxic targets-bile acids” network. The red and purple border respectively represents time-related and dose-related targets and bile acids, while the others are common to both.

We identified 14 potential STRER active components in the TCMSP database and through the literature search; these included 10 alkaloids previously reported to induce liver toxicity. To identify the hepatotoxic targets of STRER, 563 STRER targets and 502 hepatotoxic targets were merged in Cytoscape. A total of 76 intersecting genes considered to be potential STRER hepatotoxic targets were identified and imported into the IntAct, BioGRID, and MINT databases for the construction of the PPI network as shown in Figure 1A. By screening network parameters, 224 nodes were identified and overlapped with the STRER hepatotoxic targets to obtain 54 core targets; EGFR, APP, ESR1, PARP1, and MDM2 were the high ranked core targets. These potential STRER hepatotoxic targets were used to conduct GO and KEGG analyses in Metascape and the results are shown in Figures 1B,C. The top 20 GO analysis results with respect to the three terms, biological process, cellular component, and molecular function, as well as top 10 KEGG analysis findings are shown. The term “biological process” was identified as being associated with inorganic substances, the organic hydroxy compound metabolic process, and response to toxic substances. Protein serine/threonine/tyrosine kinase activity, protein serine/threonine/tyrosine kinase activity, lipid binding activity, and oxidoreductase activity were found to be associated with “molecular function”. Pathways in cancer, alcoholic liver disease, and bile secretion were among the 10 most significant pathways in the KEGG analysis.

### 3.2 Effects of STRER induced liver injury in rats

**FIGURE 2:**
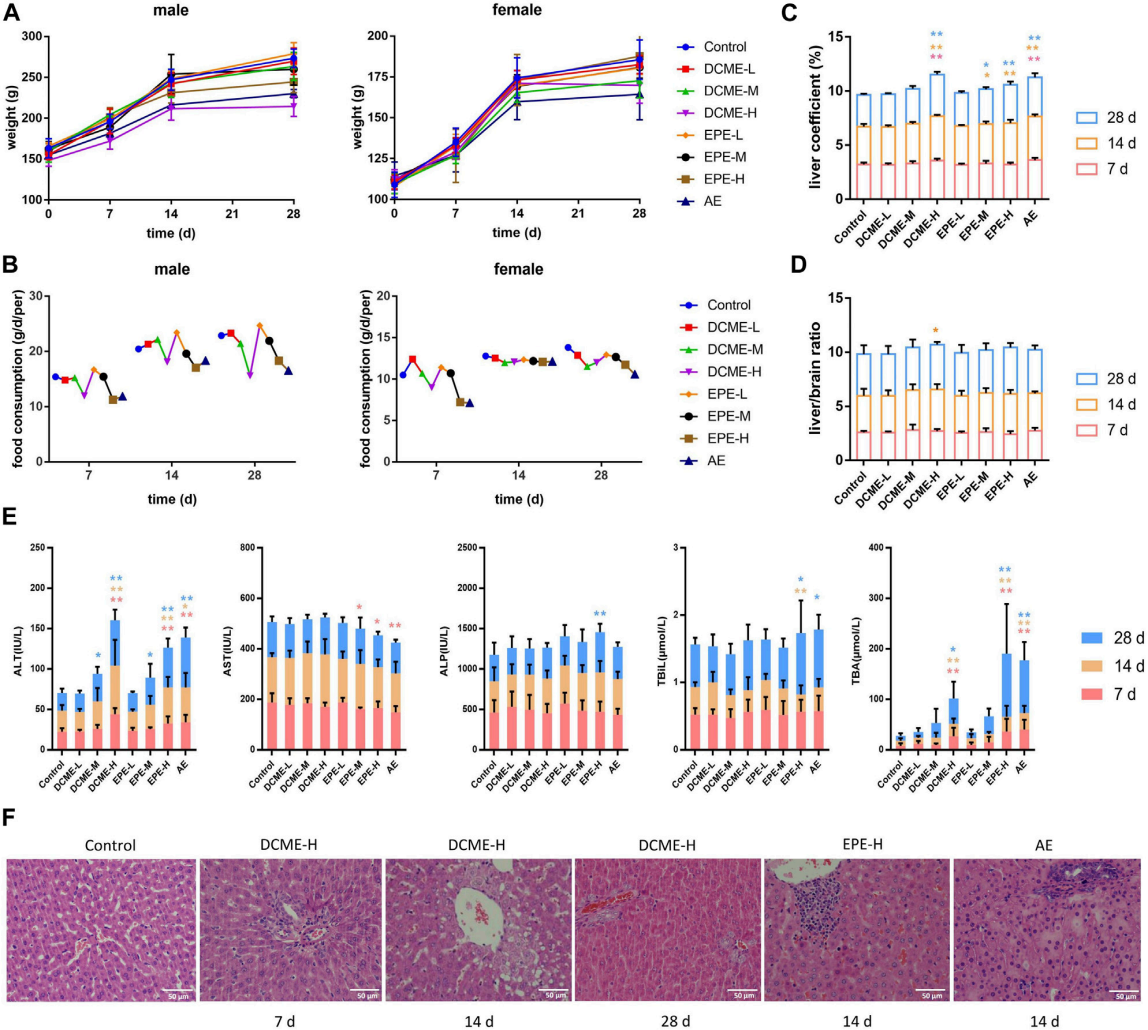
Hepatotoxic effects of different STRER extracts on rats by repeated gavage for 28 days. **(A)** Body weight, **(B)** food consumption, **(C)** liver coefficient and **(D)** liver/brain ratio of rats with administration during 28 days. **(E)** The contents of ALT, AST, ALP, TBIL and TBA in rat serums of different days. All data were presented as mean ± SD and compared with Control group. *p < 0.05; **p < 0.01. **(F)** Pathological changes of liver (HE×400). Slight biliary hyperplasia, hepatic cell micro-vacuolation and mast cell infiltration were separately found in DCME-H group of day 7 and day 14. Proliferation of bile ducts was observed from DCME-H group on day 28. Inflammatory cell infiltration was appeared in EPE-H group and AE group on day 14.

The MT and OMT contents of the DCME as determined through HPLC were 54.84% and 22.2%, which of the EPE were 1.47% and 1.98%, respectively. Salivation was observed in rats in the DCME and AE groups after treatment for 7 days and in EPE group for 14 days. The increase in weights of rats in the experimental groups was slower than those of rats in the control group, which exhibited a time-dose-effect. Food consumption in rats decreased with a decrease in the dose of extract, which was most considerably in the AE group. The weight and food consumption curves of the rats are shown in Figure 2 (A and B). After 7 days of administration, the liver coefficients of rats in the DCME-H and AE groups were considerably higher than those of rats in the control group (Figure 2C). As concerns the liver/brain ratio, there was no statistically significant difference between the different groups; however, a slight upward trend was observed in the treatment groups as compared to the control group (Figure 2D).

To determine the liver injury potential of STRER extracts, ALT, AST, ALP, TBIL, and TBA serum levels were analyzed using a biochemical analyzer to estimate the extent of liver injury. As compared to rats in the control group, rats in the DCME-H, EPE-H and AE groups exhibited a significant increase in ALT and TBA levels. ALT and TBA levels in rats in the DCME group and EPE group were found to increase with increase in the dose of the extract, and these levels were significantly different in contrast to rats in the control group. These results were statistically significant and a “dose-toxicity” effect was observed. As administration time increased, ALT and TBA levels showed a significant increasing trend in the rats of DCME-M and EPE-M groups after 14 days, as shown in Figure 2E.

The pathological method is the gold standard method for the diagnosis of liver injury. The livers of rats in the treatment groups exhibited varying degrees of inflammatory cell infiltration, bile duct hyperplasia, and hepatocyte vacuolation. After 7 days of administration, a few rats in the DCME-H group exhibited slight bile duct hyperplasia and lymphocyte infiltration. After 14 days of administration, rats in EPE-H and AE group appeared inflammatory cell infiltration and rats in DCME-H group exhibited hepatic cell micro-vacuolation. After 28 days of treatment, inflammatory cell infiltration and hepatic cell micro-vacuolation were observed in rats in the DCME-L and EPE-L groups, while bile duct hyperplasia was observed in rats in the DCME-H and AE groups. A greater number of rats exhibited pathological changes in the DCME group than in the EPE and AE group, and this number increased as administration time increased. The extent of pathological injury tended to increase with increase in DCME dose. HE staining findings are shown in Figure 2F.

### 3.3 Identification of potential metabolite of STRER induced liver injury

**FIGURE 3:**
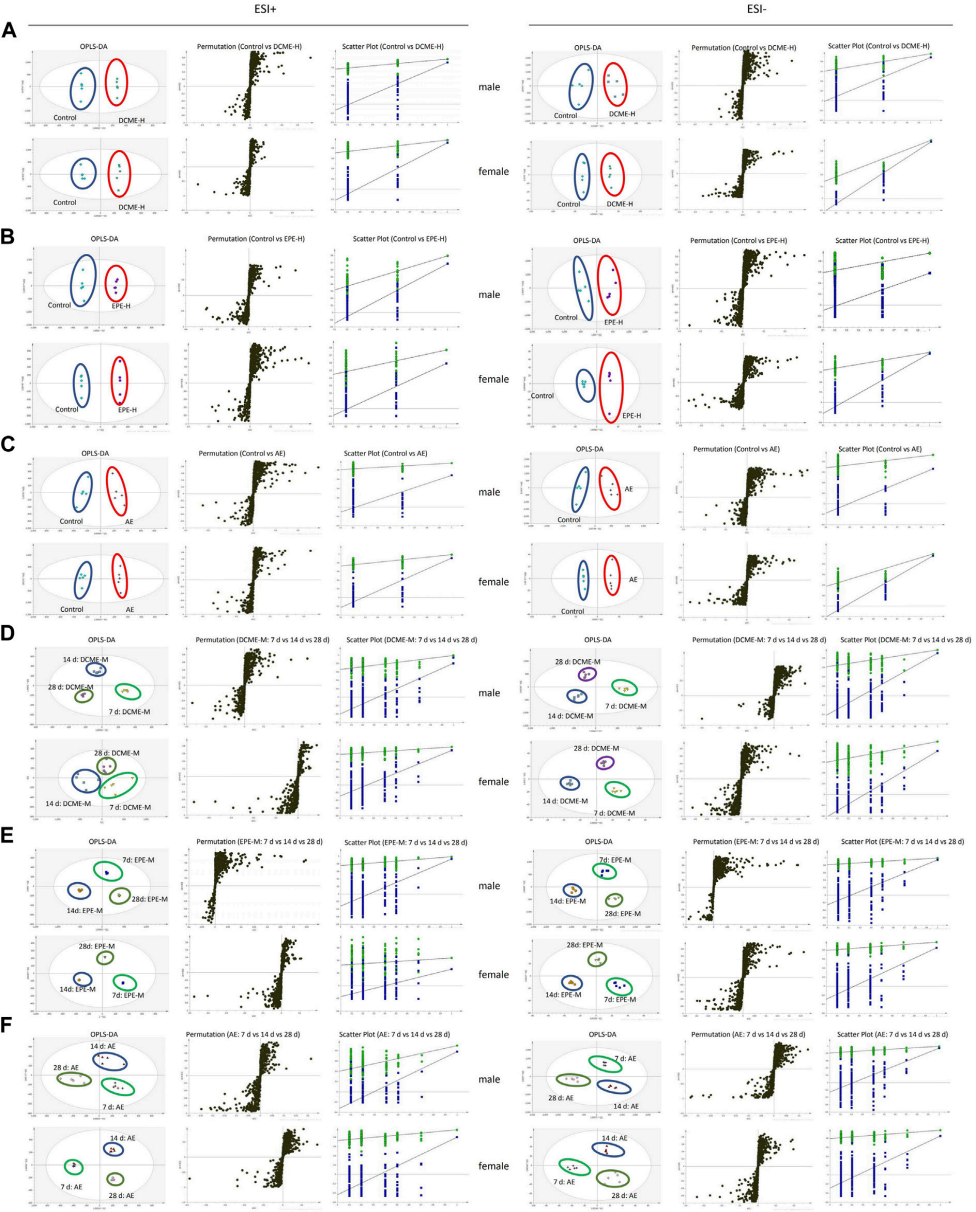
The OPLS-DA score plots, S-plots and 200-permutation test in the positive (ESI+) and negative (ESI-) modes. **(A)** Comparison of DCME-H and Control groups. **(B)** Comparison of EPE-H and Control groups. **(C)** Comparison of AE and Control groups. **(D)** Comparison of DCME-M group on days 7, 14 and 28. **(E)** Comparison of EPE-M group on day 7, 14 and 28. **(F)** Comparison of AE group on day 7, 14 and 28.

To investigate the dose-effect relationship of STRER and its alkaloids with respect to the induction of hepatoxicity, serum samples of rats in the control, DCME-L, DCME-M, DCME-H, EPE-H and AE groups were collected at day 14. Serum samples of rats in the control, DCME-M, EPE-M and AE groups were analyzed on days 7, 14, and 28 to investigate the time-effect relationship of the different treatments. Multivariate statistical analysis, including unsupervised pattern recognition analysis (PCA) and orthogonal partial least squares discriminant analysis (OPLS-DA), was performed to explore the clustering trend in metabolite profiles between the treatment groups and the control group. The PCA and OPLS-DA of the DCME-H, ETE-H and AE groups contrasted with control group performed cluster trend, and satisfactory separation was observed in both the positive and negative ion modes, indicating the high reproducibility and stability of the instrument; this also indicated that the model had a satisfactory interpretation rate. As concerns the OPLS-DA model, as shown in Figure 3, permutation tests provided evidence of its validity and applicability, and the intercept of Q2 was less than 0 for the regression line in scatter plot, indicating the prediction effectiveness of the model. Differential metabolites were identified in the METLIN database based on the molecular weights of the extracts. As shown in Table 1, the levels of 15 and 13 serum differential metabolites with dose-effect and time-effect relationships, respectively, as screened by variable importance in the projection (VIP >1) in the S-plot OPLS-DA model were significantly altered following the administration of the STRER extracts (*p*<0.05). Metabolic pathway analysis was performed using MetaboAnalyst and the findings thereof are shown in Figure 1D. These findings indicated that bile acid metabolism, the citrate cycle (TCA cycle), and amino acid metabolism were significantly interrelated with the efficacy of STRER.

**TABLE 1:** Differential metabolites with dose-time-effect induced by STRER.

No.	Metabolites	Dose	Time
1	Cholic acid	↑	↑
2	Deoxycholic acid	↑	↑
3	Glycoursodeoxycholic acid	↑	↑
4	Glycocholic acid	↑	↑
5	Taurocholic acid	-	↑
6	Chenodeoxycholic acid 3-glucuronide	-	↑
7	Taurochenodeoxycholic acid	↑	-
8	Pyruvic acid	↓	-
9	(±)-Malic Acid	↓	-
10	alpha-Ketoglutaric acid	↓	↓
11	Citric acid	↓	↓
12	Hippuric acid	↑	↑
13	Salicylic acid	↑	↑
14	DL-Tryptophan	-	↓
15	3-Indolepropionic acid	↑	-
16	L-alpha-lysophosphatidylcholine	↑	↑
17	Ethyl (4Z,7Z,10Z,13Z,16Z,19Z)-4,7,10,13,16,19-docosahexaenoate	-	↑
18	5,6-Dihydroretinoic acid	↑	-
19	(2R)-2-Hydroxy-3-(stearoyloxy)propyl 2-(trimethylammonio)ethyl phosphate (PC (18:0))	↑	-

### 3.4 Effects of bile acids in rat with administration of STRER

Based on the metabolic pathways of differential metabolites as determined through the untargeted metabolomics analysis, we focused on bile acid analysis. Liver injury induced by STRER and its alkaloids in rats was found to reduce bile secretion and affect the distribution of bile acids, and this resulted in a significant increase in serum bile acid concentrations. The levels of 8 and 15 bile acids were found to vary with increase in experimental dose and administration time, respectively.

**FIGURE 4:**
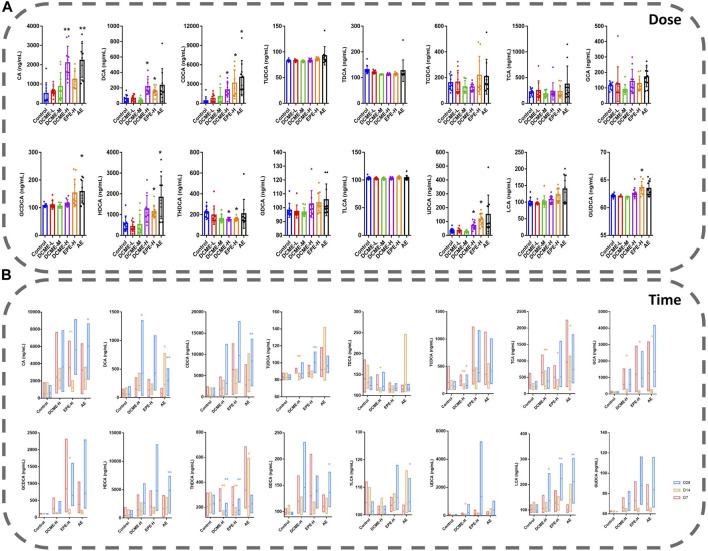
Bile acids in rat serums after multiple administration of different doses and times of STRER extracts (n = 10, mean ± SD). **(A)** Experimental groups with different doses were contrasted with control groups. **(B)** Comparison between different time points for each group. *p<0.05; **p<0.01.

After 14 days of continuous treatment, DCA, CDCA, HDCA, UDCA and GUDCA levels in the EPE-H group, CA, CDCA, GCDCA and HDCA levels in the AE group, as well as CA, DCA, CDCA and UDCA levels in the DCME-H group, were significantly elevated with *p*-value <0.05, as compared to their levels in the control group. There was a decrease in THDCA content in the DCME-H and EPE-H groups. These findings indicated that STRER extract-induced liver injury, was possibly related to bile acid secretion, especially that induced by its alkaloid fraction was dose-dependent. The results are shown in Figure 4A.

As concerns the comparison of bile acid levels after 7, 14 and, 28 days of treatment, DCA and LCA levels in DCME-H group, as well as DCA, CDCA, HDCA, TLCA and LCA levels in AE group were increased significantly as treatment time increased. The same significant elevation as UDCA level in DCME-H group, TUDCA and LCA levels in EPE-H group, CA and GDCA levels in AE group after 28 days of treatment but no time dependency. TUDCA, TDCA and TCA levels in the DCME-H group, CA, TCA, GCA and GCDCA levels in the EPE-H group, TCA, GCA and THDCA levels in the AE group showed a fluctuation that first significant decreases after 14 days and then increases after 28 days. The levels of TCDCA in DCME-H group and THDCA in DCME-H and AE group were decreased after 28 days of treatment but no time dependency. This suggested that the change in bile acid levels with increase in treatment time was caused by drug factors as shown in Figure 4B.

### 3.5 Molecular docking

The differential bile acids identified through the quantitative analysis were used to identify 146 targets in the SwissTargetPrediction database, and targets associated with STRER hepatoxicity were identified using Venn diagrams; the “compounds-hepatotoxic targets-bile acids” network was drawn using Cytoscape and is shown on Figures 1E,F. A total of 11 compounds, 16 bile acids, and 21 hepatotoxic targets were found to play an important role in STRER-induced liver injury through the dysregulation of bile acid metabolism. The associated compounds and genes were selected for molecular docking in the AutoDockTools software.

**FIGURE 5:**
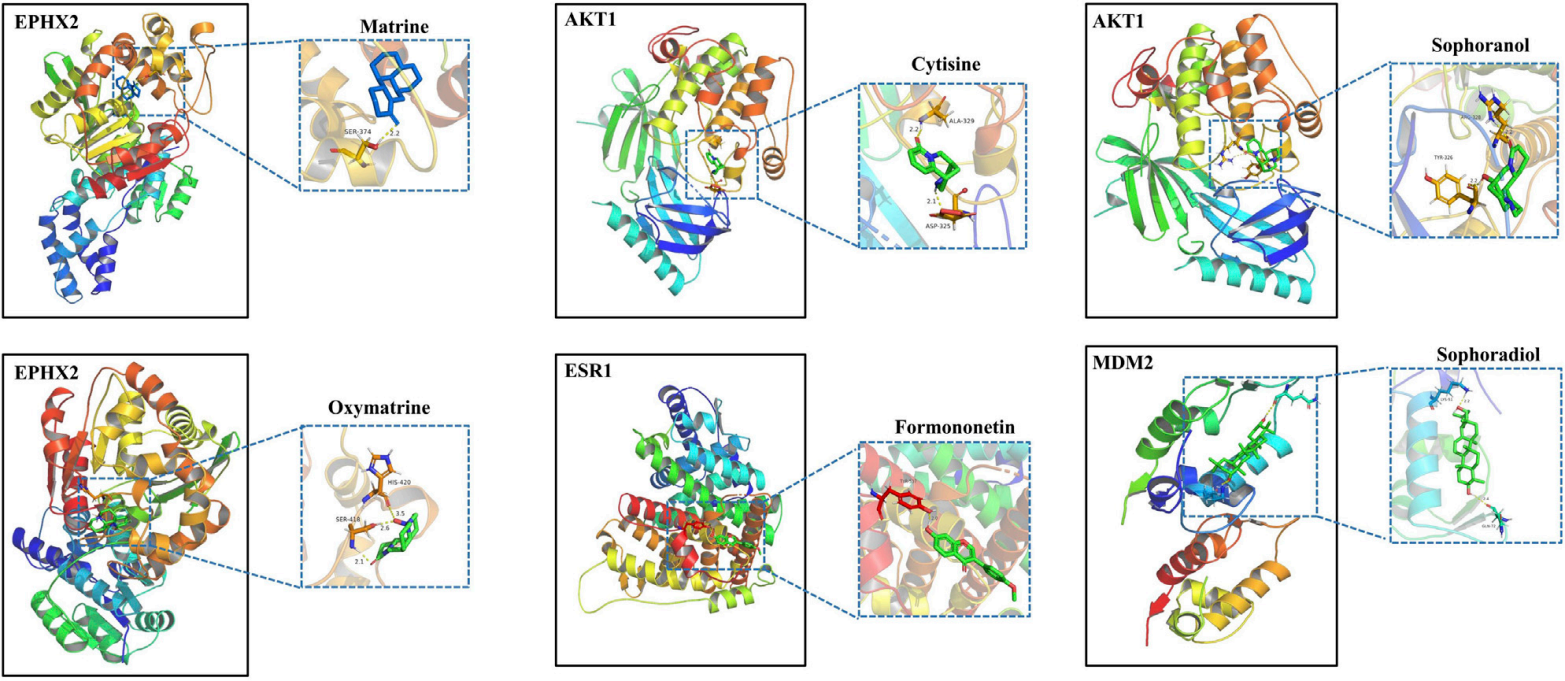
The 3D interaction diagrams of MT, OMT, cytisine, sophoranol, formononetin and sophoradiol with related key targets.

To further investigate the possibility of interactions between STRER and key targets in the bile acid pathway, we carried out molecular docking analyses. The docking scores between 21 pairs of compounds and targets were less than −5 kcal/mol, suggesting that the corresponding compounds and targets obtained by virtual screening had high binding affinities. The detailed results are shown in Table 2. Binding poses and binding sites for six pairs of compound-target interactions are shown in Figure 5.

**TABLE 2:** The docking scores between key targets of bile acids and STRER compounds (kcal/mol).

Target	PDB ID	Compound	Binding score (kcal/mol)
EPHX2	5AM2	Matrine	−6.64
		Oxymatrine	−6.26
ACE	5AMB	Sophoranol	−5.35
AKT1	7NH5	Cytisine	−5.78
		Sophoranol	−6.2
AR	2AM9	Sophoranol	−5.34
		Oxymatrine	−5.65
		Sophoradiol	−6.7
		Formononetin	−5.11
CES2	5A7F	Sophoradiol	−6.53
ESR1	6SBO	Sophoradiol	−6.19
		Formononetin	−5.27
G6PD	6E08	Oxymatrine	−5.65
		Sophoradiol	−5.88
HMGCR	1DQA	Sophoradiol	−5.64
MDM2	6Q9L	Sophoradiol	−6.73
NR1H3	5HJS	Sophoradiol	−5.76
NR1H4	3BEJ	Sophoranol	−6.11
NR1I3	1XVP	Sophoradiol	−5.74
TNF	5UUI	Formononetin	−5.13
VDR	1DB1	Sophoradiol	−6.51

## 4 Discussion

We found that STRER induced liver injury through changes in bile acid metabolism caused by its alkaloid constituents; we used a combination of SysT and metabolomics to determine the molecular mechanisms underlying these effects. First, through the SysT analysis, were screened out 14 STRER compounds and 76 associated hepatoxic targets. EGFR, APP, ESR1, PARP1, and MDM2 exhibited high frequency links in the PPI network, indicating that they possibly played important roles as targets in the hepatotoxicity mechanism of STRER. Second, after administering the different treatments in rats for 28 consecutive days, the alkaloid-rich STRER DCME induced significant hepatotoxicity in rats as evidenced through the analysis of body weight, food consumption, serum biochemical parameters, and pathological findings. Rat body weights slowly increased after 7 days of treatment with the DCME and EPE, and a “dose-time-toxicity” relationship was observed. A partial decrease in food consumption resulted in weight deficit aside from the effects of the drug. ALT and TBA levels in the DCME-H, EPE-H and AE groups were significantly higher than those in the control group. ALT and AST are widely used as sensitive hepatic biochemical parameters in the diagnosis of liver toxicity; they are used for the evaluation of toxicity in both clinical and non-clinical settings (Ramaiah, 2007). ALP, TBIL, bile acids, and gamma glutamyltransferase (GGT), which are also biomarkers of liver function, were used in combination to diagnose liver injury, particularly bile duct damage (Ozer et al., 2008). Liver pathological changes, hepatocyte necrosis, bile duct injury, and inflammation, which are associated with the extent of liver damage, gradually increased with increase in treatment time and extract dose in the experimental groups. The results of biochemical and pathological analysis in AE group were more obvious than those in DCME and EPE groups, which was same as the study of hepatotoxicity induced by extracts of STRER in zebrafish: the water extract > dichloromethane extract > ethanol sedimentation extract (Liu et al., 2017). It is speculated that other components in STRER played a synergistic role with alkaloids to aggravate liver toxicity in rats. These findings indicated that STRER have a certain degree of liver toxicity, and should be used cautiously in clinical settings, especially in patients with liver dysfunction.

As concerns metabolomics, using an omics method that identifies all low-molecular weight metabolites in biological systems during specific physiological periods, with simultaneous qualitative and quantitative analyses, we identified 15 and 13 metabolites with dose-effect and time-effect relationships, respectively. Of note, differential metabolites that participate in primary bile acid biosynthesis and the TCA cycle were largely from the administration factors, indicating potential metabolic responses during STRER-induced hepatic injury. This finding was consistent with the findings of a previous SysT KEGG analysis for STRER hepatotoxic targets, important one of which was bile secretion pathway. In DILI patients, serum bile acids were the main metabolites with altered levels and were the most significantly and consistently associated with the severity of DILI (Zhao et al., 2022).

Our findings showed that bile acid metabolism is possibly associated with STRER hepatotoxicity; however, the functional characteristics and mechanism of this association are unclear. We evaluated the rat serum contents of 16 kinds of bile acids, the levels of CA, DCA, CDCA, HDCA, UDCA, GUDCA and GCDCA were found to significantly increase with administration of DCME-H, EPE-H and AE. Bile acids are classified into hydrophilic and hydrophobic bile acids based on their chemical structures. Hydrophobic bile acids can dissolve cell membrane lipids; CA, CDCA, and DCA are free hydrophobic bile acids with significant hepatotoxic effects, with DCA being one of the most toxic bile acids. The accumulation of DCA may lead to hepatocyte mitochondrial damage, increase in reactive oxygen species levels, and hepatocyte apoptosis and necrosis (Wang et al., 2021). THDCA levels were found to decrease in a dose-time-dependent manner following the administration of DCME and AE. Primary bile acids exist mainly in ionized forms combined with glycine (75%) and taurine (25%). This coupling increases the hydrophilicity of bile acids and reduces their cytotoxicity (Faustino et al., 2016). Coupled bile acids can only enter cells, where they induce intracellular toxicity, through transporters (Schadt et al., 2016). By analyzing serum bile acid levels in patients with DILI, it was shown that GCA, TCA, TUDCA, GCDCA, GCDCS, and TDCA could be useful markers for DILI diagnosis and early liver injury prediction. CDCA, DCA, and LCA can predict the severity of DILI and play a role in its pathogenesis (Ma et al., 2019). In this study, CA and DCA levels in rat serum significantly increased with increase in DCME dosage. DCA, CDCA, HDCA, TLCA and LCA levels significantly increased with the administration time of DCME and AE, and these levels were related to the degree of liver injury. Bile acid metabolic disorders can induce significant changes in blood bile acid content following liver damage. Therefore, biomarkers of bile acid metabolic pathways are important indicators in the diagnosis and treatment of liver diseases (Chang et al., 2017). Cholestatic liver injury was found to be mainly reflected by changes in bile acid metabolism; TCA, GCA, GCDCA, and GDCA may be sensitive biomarkers for liver injury induction (Zhang et al., 2021). In an induced rat liver injury model, bile secretion was found to decrease with increase in CDCA, DCA, UDCA, CA, GCDCA, GDCA, GCA, TCDCA, TUDCA, and TCA content (Wang et al., 2021). In the experimental groups, there was a significant increase in DCA and UDCA levels in the DCME-H group. GCDCA, HDCA, and GDCA levels significantly increased in the AE group. As treatment time increased, CA, DCA, CDCA, TUDCA, GCA, GCDCA, HDCA, UDCA, GDCA, and GUDCA levels in rats in the experimental groups increased to different degrees. Accumulated bile acids irritate bile duct cells and lead to bile duct hyperplasia, which is characteristic of cholestatic liver disease (Sato et al., 2018). Bile duct hyperplasia, hepatocyte injury, and inflammatory cell infiltration were observed in rats in the experimental groups. A previous study suggested that STRER might induce mixed liver injury, with hepatocyte injury and cholestasis simultaneously occurring (Wang et al., 2019).

To further investigate the potential mechanisms and targets of STRER-induced hepatotoxicity in terms of bile acid metabolism, we analyzed the “compounds-hepatotoxic targets-bile acids” network and extracted 11 key compounds, 16 bile acids, and 21 hepatotoxic targets. After molecular docking, we confirmed that six important compounds and 14 potential targets played important roles in STRER-induced liver injury in relation to bile acid metabolism. MT, OMT, sophoradiol, sophoranol, cytisine, and formononetin were found to be potentially hepatotoxic components. Bifunctional epoxide hydrolase 2 (EPHX2), androgen receptor (AR), Vitamin D3 receptor (VDR), 3-hydroxy-3-methylglutaryl-coenzyme A reductase (HMGCR), glucose-6-phosphate dehydrogenase (G6PD), and Estrogen receptor (ESR1) were considered to be core targets. GCA, DCA, CDCA, UDCA, and LCA were core metabolites significantly associated with STRER hepatoxicity. EPHX2 plays a role in xenobiotic metabolism by degrading potentially toxic epoxides. It has been reported to be under-expressed in liver cancer tissues and cell lines (Zhan et al., 2021), and encodes soluble epoxide hydrolase (sEH), which is a key regulator of hepatic inflammation and injury (Schuck et al., 2014). Steroid sex hormones may induce the occurrence and development of hepatocellular carcinoma through relevant receptors in the liver; among these receptors, AR negatively correlated with the degree of liver injury (Mishkin et al., 1989; Li et al., 2019). ESR1 was shown to play an important role in liver metabolism and was identified as the main regulator of the expression of several CYP enzymes, thereby affecting drug metabolism (Khristi et al., 2019; Collins and Wang, 2021). The inhibition of ESR1 was found to be significantly associated with the recurrence and progression of intrahepatic cholangiocarcinoma (Li et al., 2021). VDR is involved in the metabolism and transport of bile acids and plays a protective role in liver. Its absence was shown to trigger calpain activation and E-cadherin cleavage, thereby exacerbating cholestatic liver injury (Firrincieli et al., 2013). HMGCR was shown to be significantly involved in cholesterol metabolism in liver and affect bile acid transformation and output (Loh et al., 2018; Chen et al., 2021). G6PD, which induces cancer and promotes cancer progression, was found to be activated by long-term exposure to bile acids (Munemoto et al., 2019), and its absence was shown to induce acute viral hepatitis and lead to liver failure in severe cases (Kamani et al., 2020).

MT and OMT are quality index constituents of STRER as stipulated in the Chinese Pharmacopoeia. Only a small amount of MT is detected in blood following the intravenous administration of OMT (Wu et al., 2006); however, high MT concentrations are detected following oral OMT administration (Fan et al., 2013). As the main component that is absorbed into blood circulation, MT plays a major role in the pharmacodynamic and toxic effects of STRER following its oral administration. CYP liver isoenzyme subtypes, especially CYP3A4, were found to convert OMT to MT through a reduction reaction (Liu et al., 2015). Experimental analyses of the hepatotoxic effects of STRER have established alkaloids as the substances responsible for its toxic effects, thereby limiting its application and development (Liu et al., 2017; Wang et al., 2017; Gu et al., 2019). It was suggested that MT may be the main STRER toxic component that induces liver injury. Furthermore, STRER-induced DILI was shown to be driven by several other matrine- and cytisine-type alkaloids (Qiu et al., 2018; Yu et al., 2018). When we compared the liver injury-inducing effects of DCME and AE of STRER, AE seemed to display higher toxicity than DCME. Therefore, we speculated that a few other ingredients, other than alkaloids, synergistically enhanced STRER toxicity. The hepatotoxic material basis and mechanism of STRER still need to be further investigated and described, and this study provides a reference for future related studies.

## 5 Conclusion

This study employed an integrated strategy using serum metabolomics and systems toxicology to explore the toxic mechanism and material basis of STRER-induced hepatotoxicity in relation to bile acid metabolism. STRER alkaloids were the main components responsible for its toxic effects. Differential metabolites and related pathways were identified through the metabolomics analysis. Through the quantitative analysis, 16 significantly altered bile acids, as well as STRER-related targets and compounds, were identified. Molecular docking further validated the binding of 14 targets and six compounds. The data and theoretical support provided by this study laid a foundation for in-depth studies into the hepatotoxic mechanisms of STRER.

## Data Availability

The original contributions presented in the study are included in the article/Supplementary Material, further inquiries can be directed to the corresponding authors.
